# Electrical impedance spectroscopy in plant cold resistance: a review

**DOI:** 10.7717/peerj.20596

**Published:** 2026-01-19

**Authors:** Zhanyu Wang, Shuoyu Di, Xinyi Zhang, Jia Yang, Chunyun Zhou, Xinmin Deng, Yu Meng, Luping Ma

**Affiliations:** 1 Hebei Agricultural University, Baoding, China; 2 Liyuan State-owned Forest Farm, Xiaogan, China

**Keywords:** Electrical impedance spectroscopy, Plant cold tolerance, Electrolyte leakage, Equivalent circuit model, Non-destructive testing

## Abstract

Low-temperature stress compromises the integrity of plant cell membranes, leading to lipid phase transitions and increased membrane permeability, which subsequently induce physiological damage. However, conventional methods for assessing cold resistance, such as relative electrolyte leakage measurement, growth recovery tests, and LT50 determination, are limited by their highly destructive nature, time-consuming procedures, or insufficient sensitivity. Electrical impedance spectroscopy (EIS), a non-destructive and efficient electrophysiological technique, has emerged as a valuable tool for evaluating cold resistance and screening cold-tolerant plant varieties. By applying multi-frequency alternating current to plant tissues and measuring the resulting impedance responses, EIS enables the extraction of key parameters such as extracellular resistance, intracellular resistance, and cell membrane capacitance. These parameters collectively reflect the structural integrity and physiological condition of cells from multiple perspectives. Notably, under low-temperature stress, plant genotypes with varying degrees of cold resistance exhibit distinct impedance spectral characteristics, allowing EIS to efficiently discriminate cold tolerance among different varieties or treatments. This review summarizes recent advances in EIS-based research on plant cold resistance, covering its underlying electrical principles, equivalent circuit models, and biophysical mechanisms. It also outlines practical applications, including the screening of cold-tolerant woody and herbaceous plants, as well as integration with traditional assessment methods, while highlighting the advantages of EIS in terms of accuracy, universality, and real-time monitoring. Furthermore, the review addresses key challenges such as species specificity, model standardization, and data analysis, and proposes future research directions, including integration with artificial intelligence, development of portable devices, and establishment of standardized stress resistance databases.

## Introduction

Low-temperature stress is a major limiting factor for crop growth and agricultural production. Low-temperature stress induces a liquid-gel phase transition in membrane lipids, diminishing fluidity and causing electrolyte leakage (Aslam et al., 2022; Tian et al., 2022). This membrane failure initiates cellular damage pathways, such as dehydration, metabolic arrest, and oxidative stress, leading to plant death. As countermeasures, plants have evolved adaptive responses, including osmolyte accumulation, antioxidant enzyme activation, and increased lipid unsaturation, to preserve membrane function and cellular homeostasis (Zhang et al., 2020; Zhao et al., 2020; Guo et al., 2022; Zuo et al., 2022; Habibi et al., 2022; Li et al., 2024). However, cold resistance varies widely among species and varieties, highlighting the urgent need for accurate assessment methods to screen and cultivate cold-tolerant varieties in horticulture, forestry, and agricultural breeding (Moura et al., 2017; Adhikari et al., 2022). This would help improve crop survival and yield in cold climates.

Traditional methods for assessing plant cold tolerance include measuring electrolyte efflux rates, observing tissue browning, conducting growth recovery tests after low-temperature acclimation, and determining LT_50_ (Wiering et al., 2018; Valverde et al., 2024). For example, the electrolyte efflux method indirectly quantifies membrane damage by measuring ion leakage in the culture medium after low-temperature treatment. Combined with logistic equation fitting of the temperature-injury curve, this method can calculate the LT_50_ value and is commonly used in comparative cold tolerance studies of crops such as citrus fruits, grapes, tea trees, and roses (Król, Amarowicz & Weidner, 2015; Samarina et al., 2020; Gutiérrez-Villamil, Balaguera-López & Álvarez-Herrera, 2023; Wang et al., 2023). However, these methods are often cumbersome, time-consuming, and destructive, making it difficult to monitor the same plant continuously and in real time. Moreover, traditional methods cannot provide detailed information on structural changes at the cellular level, which is a limitation when screening large-scale germplasm resources for cold tolerance.

Electrical impedance spectroscopy (EIS) is a non-destructive electrophysiological technique that offers a promising alternative for studying plant cold tolerance (Ma et al., 2023). EIS measures the response of plant tissues to wide-band alternating current at various frequencies, enabling sensitive detection of changes in cell membranes, and intracellular and extracellular electrolytes under cold stress (Okajima & Sugiyama, 2023; Okajima, Nakagawa & Sugiyama, 2023). Compared to traditional methods, EIS offers several advantages: EIS does not damage plant tissue, allowing multiple measurements on the same plant and enabling real-time monitoring while the plant is still alive. A single test typically takes only a few minutes, making it ideal for high-throughput screening of large sample sets (Aouane et al., 2021; Caeiro, Canhoto & Rocha, 2025; Zhang et al., 2025); EIS provides multiple electrical parameters, including extracellular resistance (R_e_), intracellular resistance (R_i_), and membrane capacitance (C_m_), offering a more comprehensive view of cell structure and function than traditional indicators like REL (Tang et al., 2024; Wulandari, Zarkasi & Nurhanafi, 2024); EIS parameters are highly sensitive to changes in membrane phase and electrolyte exudation. This sensitivity allows EIS to detect physiological changes before visible symptoms appear, providing an early warning system.

In recent years, numerous studies have applied EIS to evaluate cold tolerance in various plant species. For instance, Qian et al. (2021) used electrical impedance tomography (EIT), an advanced form of EIS, to assess the cold resistance of rose branches. Their results showed that impedance values measured by EIT decreased significantly with decreasing temperature, and the LT_50_ of the branches could be calculated using the Logistic model. EIT results also demonstrated a high linear correlation with cold tolerance data obtained using the traditional electrolyte efflux method (r ≈ 0.93), confirming the reliability of EIS for non-destructive cold tolerance assessment. Another study by Tiejun et al. (2024) examined the impedance response of rice seedlings under low-temperature stress, finding that EIS parameters could sensitively reflect the extent of cell damage and recovery following freezing.

This review synthesizes the technical principles, equivalent circuit models, and applications of EIS in plant cold-stress studies. It examines the biophysical and physiological mechanisms of cold tolerance revealed by EIS and critically analyzes the technique’s advantages and limitations. Future directions for integrating EIS with artificial intelligence, portable devices, and standardized databases are also discussed. Aimed at researchers in plant physiology, agricultural engineering, and breeding, this work seeks to provide a technical foundation and methodological reference for applying EIS in cold-resistance research and germplasm screening.

## Survey methodology

To provide a comprehensive and critical overview of the application of EIS in plant cold tolerance research, a systematic and reproducible literature search was conducted. The literature search was conducted across multiple core academic databases and publishing platforms, including the multidisciplinary citation databases Web of Science Core Collection and Scopus, the authoritative engineering database EI Compendex, and major publishing platforms such as Science Direct, SpringerLink, and Wiley Online Library. The primary search terms included: “electrical impedance spectroscopy”, “EIS”, “plant”, “cold tolerance”, “cold resistance”, “freezing stress”, “electrolyte leakage”, and “equivalent circuit model”. These terms were used in various combinations to maximize the retrieval of relevant studies.

The systematic screening process, summarized in Table 1, was conducted in several stages. Initially, 6,144 records were identified. After removing 3,320 duplicates and 87 records deemed ineligible by automation tools, 2,737 records were retained for title and abstract screening. At this stage, studies were evaluated against predefined inclusion and exclusion criteria. The inclusion criteria covered original research applying EIS in plant low-temperature stress studies, investigations linking EIS parameters to plant physiological status, and English-language peer-reviewed articles. Exclusion criteria included non-plant applications and non-peer-reviewed publication types such as conference abstracts and books. In total, 2,333 records were excluded: 2,154 due to mismatched research topics and 179 due to non-conforming publication types. This resulted in 404 articles undergoing full-text review.

**Table 1:** Literature screening process and exclusion criteria.

Screening stage	Operational description/Exclusion reason	Records excluded	Records remaining
Identification	Web of Science	1,803	6,144
	Scopus	2,172	
	EI Compendex	1,102	
	Science Direct	617	
	Springer	338	
	Wiley	112	
Initial screening	Duplicate records	3,320	2,737
	Records flagged as ineligible by automation tools	87	
	Focus on EIS applications outside of agronomy	2,154	404
Title/Abstract screening	Ineligible publication type (*e.g*., conference abstracts, books)	179	
	Research content deviated from the core focus of this review	117	93
Full-text assessment	Failed to establish a valid correlation between EIS and plant physiological indicators	89	
	Incomplete methodology description or unusable/extractable data	103	
	Focus on other plant stress resistance rather than cold resistance	58	11
Core analysis	Lacked in-depth analysis linking electrical impedance parameters to resistance phenotypes/mechanisms	24	

During the full-text assessment, each study was evaluated for its relevance to plant cold resistance, robustness in correlating EIS parameters with physiological indicators, and methodological completeness. A total of 309 articles were excluded: 117 for deviating from the core research focus, 89 for insufficient EIS-physiology correlation, and 103 for incomplete methods or unavailable data. Ultimately, 93 studies met all inclusion criteria and were included in the review. From these, a subset of 11 studies that were most closely aligned with the core theme of EIS in plant cold resistance was selected for in-depth feature extraction and synthesis, as summarized in Table 2. To ensure comprehensive coverage, backward citation tracking of key references was also performed to identify additional relevant studies not captured in the initial search.

**Table 2:** Summary of core studies on EIS application in plant cold resistance.

Plant material	Stress conditions	Key EIS parameters	Physiological indicators	Key findings
*Pinus bungeana* (shoot/needle)	Monthly tests (4 °C to −70 °C)	R_e_, τ, β	REL, LT_50_	1. EIS (with/without freezing) effectively assesses cold resistance, correlating well with traditional methods. 2. Without freezing, τ (shoot) and β (needle) can predict cold resistance. 3. EIS significantly shortens detection time.
*Betula pendula* (stem)	Seasonal tests (4 °C to −40 °C)	R_i_, R_e_, τ, ψ	LT_50_, REL, DT_10/50_	1. Without freezing, R_i_ and R_e_ positively correlate with LT_50_. 2. Shoot tip water content stabilizes as cold resistance increases. 3. REL can predict DT_10_, LT_50_ can predict DT_50_.
*Daucus carota* (root)	Low-Temperature Blanching (50/60°C)	C_m_, R_i_, R_e_	K^+^ leakage, pectin content, firmness	1. 60 °C causes more severe membrane rupture (C_m_↓, R_i_↑) and K^+^ leakage. 2. 60 °C treatment results in better softening inhibition and more significant cell wall modification.
*Oryza sativa* (leaf)	Low-temperature stress (0 °C to −10 °C)	R_e_, R_i_, τ	REL, Injury rate	1. EIS spectrum morphology indicates freezing injury severity. 2. R_e_ and τ decrease, R_i_ increases before death. 3. EIS parameter trends align with REL/Injury Rate changes.
*Euonymus japonicus* (stem)	Seasonal tests (−4 °C to −40 °C)	R, R_i_, R_e_, τ, ψ	LT_50_	1. Non-frozen EIS parameters significantly correlate with LT_50_ (R is best). 2. After freezing, R_e_, τ, R_i_ highly correlate with LT_50_ (R_e_ is best).
*Pinus sylvestris* (stem)	Controlled freezing (0 °C to –130 °C)	τ, R_i_	Frost Hardiness (FH), DM	1. τ is the best predictor for frost hardiness and can differentiate provenances. 2. R_2_ shows no direct relationship with cold resistance.
*P. koraiensis* & *P.simonii* (stem)	Freezing (−10 °C to −80 °C)	R_e_, R_i_, τ	LT_50_, MC	1. Freezing duration has the greatest impact on LT_50_, followed by MC. 2. R_e_ is the best parameter for estimating LT_50_. 3. Machine learning models (RF/SVM) predict LT_50_ with high accuracy.
*Rosa Floribunda* (stem)	Freezing (−8 °C to −20 °C)	EIT value	LT_50_, REL	1. EIT value decreases with temperature, correlating with freezing injury. 2. EIT value can be used to calculate LT_50_ and significantly correlates with REL results (r = 0.93).
*Salix viminalis* (shoot)	Natural acclimation, Controlled freezing	R_i_, R_e_, τ, ψ	LT_50_, DW, Fatty Acid Composition	1. R_i_ correlates best with cold resistance, increasing from 1–2 Ωm to ~12 Ωm after acclimation. 2. R_i_ positively correlates with 18:2 and negatively with 18:3. 3. DW is less reliable for prediction than R_i_.
*Malus domestica* (root)	Freeze-thaw (−3 °C to −9 °C)	R_e_, τ, R	Cell Viability (TTC)	1. EIS detects viability changes at −3 °C, while TTC only at −9 °C. 2. R_e_ and τ decrease with increasing cold stress. 3. EIS provides information on hardiness level and freeze-thaw history.
*Pinus bungeana* (stem/needle)	Different freezing temperatures	τ, R, R_e_	REL, LT_50_	1. τ significantly correlates with cold resistance, enabling rapid assessment. 2. EIS responses differ between tissues, reflecting structural differences.

**Note:**

These studies were included for in-depth synthesis following the systematic screening process detailed in Table 1.

R, Total tissue resistance; R_e_, extracellular resistance; R_i_, intracellular resistance; τ, relaxation time; ψ, distribution coefficient; C_m_, cell membrane capacitance; β, CPE parameter; EIT, electrical impedance tomography. LT_50_, median lethal temperature; REL, relative electrolyte leakage; DT_10/50_, 10%/50% tissue damage temp.; MC, moisture content; DM/DW, dry matter/weight; FH, frost hardiness.

## The principle and measurement method of EIS technology

**Figure 1:**
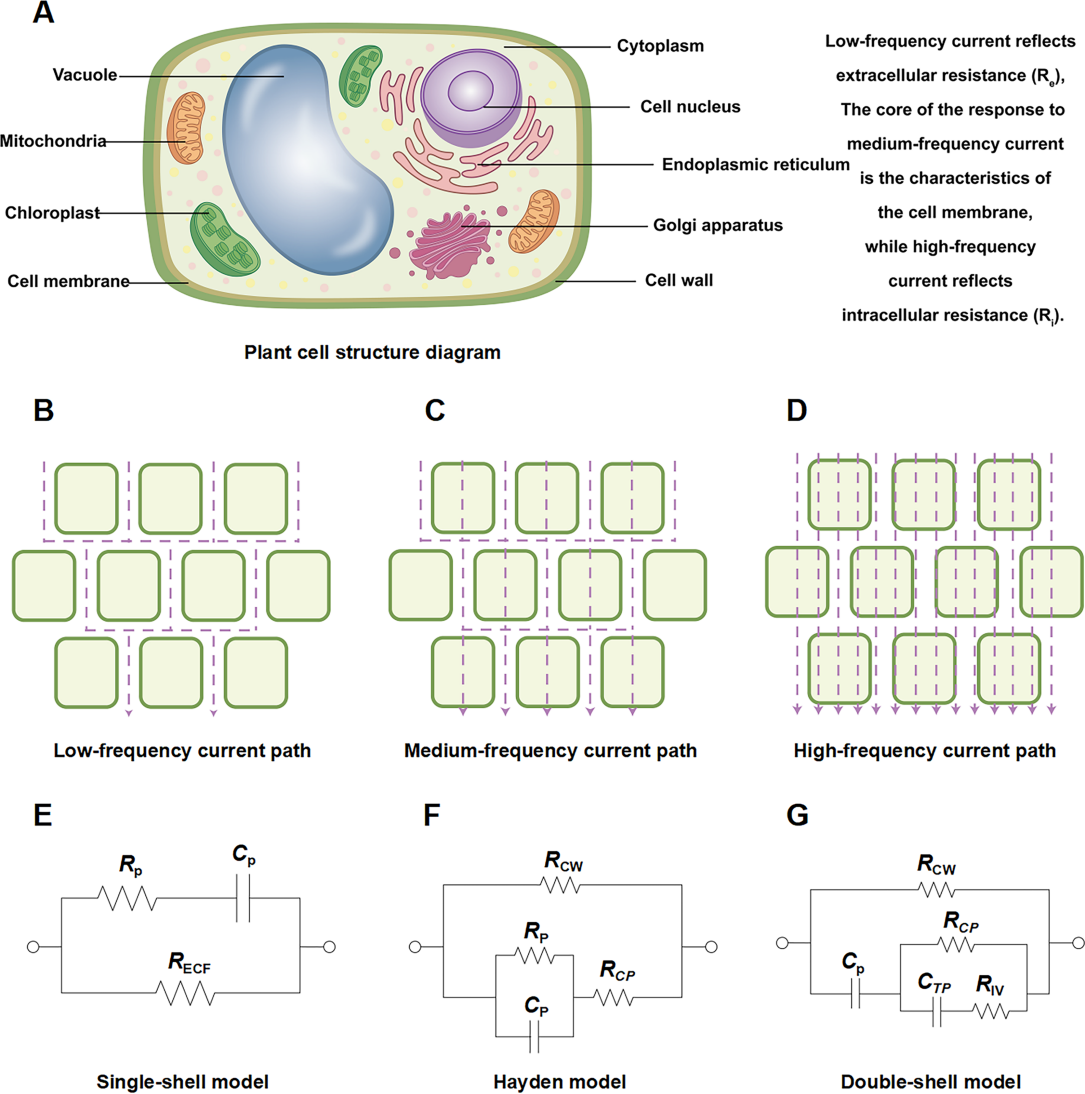
Schematic diagram of plant root cell structure, multi-frequency current pathways, and equivalent circuit models. Schematic diagram of (A) plant root cell structure, (B) low frequency current path, (C) medium frequency current path, (D) high frequency current path. Equivalent circuits for plant tissues: (E) single-shell model, P: plasma (cell wall and membrane); (F) Hayden model, CW: cell wall, CP: cell cytoplast; (G) double-shell model, TP: vacuole tonoplast, IV: inner vacuole. Note: (A–G) were created by the authors. (A) is an original figure created by the authors. (B–D) and (E–G) were adapted from Liu et al. (2021) and Serrano-Finetti et al. (2023), respectively.

Plant tissue, as a complex electrolyte-cell system, exhibits electrical impedance (Z), a composite physical quantity determined by both resistance (R) and reactance (X). Mathematically, it is expressed as Z = R + jX (Fig. 1A) (Cseresnyés et al., 2024; Pimsut et al., 2024). Here, R characterizes the obstruction encountered by electric current as it passes through the medium, while X in plant tissues primarily manifests as capacitive reactance, calculated as X = −1/ωC (where ω is the angular frequency and C is the capacitance), reflecting the ability of structures like the cell membrane to store charge (Noh, Jekal & Yoon, 2023). The current distribution pathway within the tissue exhibits significant frequency dependence (Figs. 1B–1D) (Meani et al., 2023). Under low-frequency conditions, current primarily flows through the extracellular space (*e.g*., intercellular spaces and cell walls) due to the high blocking impedance of the cell membrane. As the frequency increases, the current penetrates the cell membrane and enters the cell interior, leading to a decrease in the total impedance. Consequently, by scanning a broad frequency range (typically 1 Hz to 1 MHz), a complete impedance spectrum rich in extracellular and intracellular information can be obtained (García-Sánchez, Bragós & Mir, 2018). In this analysis, the phase angle (θ) is a key derived parameter, representing the angle between the imaginary and real components of the impedance vector. Its magnitude intuitively reflects the relative strength of the capacitive effect *vs* the resistive effect, serving as an important indicator for characterizing the dielectric properties of the tissue (Islam, Wahid & Dinh, 2018; Zhang et al., 2023). For instance, a larger phase angle indicates a stronger capacitive effect, suggesting more prominent charge-storing structures like the cell membrane, whereas a phase angle close to 0 indicates predominantly resistive impedance, which typically occurs when high-frequency current penetrates the cell interior unimpeded (Cseresnyés, Rajkai & Vozáry, 2013; Jensen et al., 2020; Tada et al., 2023).

From a cellular perspective, the impedance characteristics of plant tissues can be attributed to three core electrical parameters: extracellular resistance R_e_, R_i_, and C_m_. R_e_ represents the conductive resistance of the intercellular space, cell walls, and extracellular fluid, influenced by factors such as cell density, cell wall thickness, and extracellular ion concentration (Wang et al., 2020). R_i_ reflects the conductivity of the cytoplasm and vacuoles, affected by cell water content, internal solute concentration, and membrane integrity. C_m_ originates from the ability of the cell membrane, acting as a dielectric layer, to store charge, with its capacitance value depending on the membrane surface area, thickness, and dielectric constant (Morozumi et al., 2023). These parameters are highly sensitive to low-temperature stress. R_e_ decreases significantly upon electrolyte leakage caused by freezing injury (Fuentes-Vélez et al., 2022; Guermazi, Kallel & Kanoun, 2024). The low-temperature response of R_i_ is more complex: initial low-temperature dehydration may cause a temporary increase in R_i_, but subsequent membrane damage and solute leakage lead to a sustained decrease (Umehara et al., 2025). C_m_ often shows a reversible decrease at low temperatures due to membrane lipid phase transitions, but drops sharply or becomes undetectable upon deep freezing-induced membrane rupture (Iftikhar Hussain, El-Keblawy & Elwakil, 2018). For example, Zwiewka et al. (2015) confirmed that the R_e_ of plant tissues decreases within 1 h at −4 °C, even if the cells are not fully dead, indicating significant disruption of membrane selectivity. Monitoring the dynamic changes of R_e_, R_i_, and C_m_ aids in assessing plant cold resistance.

To transform complex impedance spectrum data into physically meaningful biological parameters, researchers commonly employ equivalent circuit models for fitting and analysis (Yao et al., 2020). Among these, the single-dispersion Cole model (Fig. 1E) is the most classic. It consists of a series resistor (corresponding to R_e_) and a parallel resistor-capacitor unit (corresponding to R_i_ and C_m_, respectively), effectively describing the impedance behavior of most herbaceous tissues. Parameters like R_e_, R_i_, and C_m_, which have clear biological significance, can be extracted by fitting impedance arc plots (Cole-Cole plots) (Watanabe et al., 2017, 2018; Ammar et al., 2023). For structurally more complex tissues (*e.g*., woody plants), it may be necessary to introduce double-dispersion or multi-dispersion models (Fig. 1G) to describe two or more relaxation processes. For instance, semicircular relaxation arcs at high and low frequencies correspond to the electrical responses of the cell membrane and the cell wall/intercellular space, respectively (Ghoneim et al., 2021; Serrano-Finetti et al., 2023). However, due to the inherent heterogeneity of biological tissues (*e.g*., membrane surface roughness, uneven component distribution), their capacitive behavior often deviates from the ideal state. To more accurately describe this non-ideal dielectric behavior, researchers often use variant models, with the Hayden model being a typical representative (Fig. 1F). The model replaces the ideal capacitor (C) with a Constant Phase Element (CPE), whose impedance is given by Z_CPE = 1/(Q(jω)^n), where Q is the CPE coefficient and n is the dispersion index (0 ≤ n ≤ 1). When n = 1, the CPE reduces to an ideal capacitor; when n < 1, it reflects the dispersion effect of the system, and a value closer to 1 indicates behavior closer to an ideal capacitor (Ando, Mizutani & Wakatsuki, 2014; Elwakil, Al-Ali & Maundy, 2021). In plant low-temperature stress studies, using the Hayden model for fitting allows for more accurate extraction of parameters related to membrane state, as low-temperature-induced membrane lipid phase transitions and structural damage significantly affect the n value, thereby providing an additional dimension of information for assessing membrane stability. Through model fitting, besides obtaining basic parameters like R_e_, R_i_, and C_m_ (or CPE parameters), a key indicator, namely the relaxation time constant (τ), can also be derived. In the single-dispersion model, τ ≈ R_i_ · C_m_ reflects the response speed of the cell membrane RC network to external electrical stimuli (Puzenko, Ishai & Feldman, 2010). In cold resistance research, a longer τ value typically predicts a more stable cell membrane structure and is an important basis for evaluating cultivar cold tolerance (Luo et al., 2024).

In measurement practice, a precise electrode system is fundamental for obtaining high-quality EIS data (Basak & Wahid, 2023). The geometric shape and material selection of the electrodes directly determine the electric field distribution pattern, the sensitivity of the measurement area, and data accuracy. Common electrode shapes include: (1) Needle electrodes, typically made of stainless steel or platinum-iridium alloy, with a high surface-area-to-volume ratio, can be inserted into the plant tissue interior (*e.g*., stem xylem) for point source or localized measurement, but their results are significantly influenced by insertion depth and direction (Postic & Doussan, 2016; Liu et al., 2021); (2) Plate or clamp electrodes, often made of gold-plated or silver/silver chloride-coated metal plates, form enveloping contact with columnar structures like plant stems or petioles, obtaining average impedance information across the entire cross-section, offering better representativeness (Bajgar et al., 2016; Tada & Inoue, 2018); (3) Parallel plate electrodes are suitable for studies on excised tissue sections, generating a uniform electric field conducive to theoretical calculations and model validation. Regarding materials, ideal electrodes should possess chemical inertness, low contact resistance, and minimal polarization impedance. Noble metals and their coatings (*e.g*., gold, platinum, silver/silver chloride) are widely used due to their excellent conductivity and corrosion resistance, effectively reducing polarization effects caused by charge accumulation at the electrode-electrolyte interface. This effect is particularly significant in low-frequency measurements and can severely distort data. Stainless steel electrodes, while lower cost and robust, are more prone to polarization and are often used in screening studies where high precision is not required. In specific measurement procedures, plant tissue samples (*e.g*., leaves, stem segments) are placed between the aforementioned electrodes, and a small AC signal is applied for frequency scanning (Zhang et al., 2024). For instance, researchers have developed a four-electrode clamping device attached to plant stems, enabling *in situ*, non-destructive monitoring of plant physiological status (Serrano-Finetti et al., 2023).

## EIS characterization of cold resistance mechanism

The development of plant cold resistance involves complex regulation at the physiological, biochemical, and molecular levels. Changes in EIS parameters serve as physical manifestations of these internal mechanisms. The application of EIS in plant cold resistance research began with the pioneering work of Repo et al. (2000) on *Pinus sylvestris*, which first systematically established correlations between EIS parameters and the degree of cold acclimation as well as membrane stability in plants, laying the foundation for this field. Today, EIS has become an essential tool for elucidating the physiological mechanisms of plant responses to low-temperature stress by monitoring dynamic changes in key electrical parameters such as R_e_, R_i_, C_m_, and τ.

Under low-temperature stress, membrane phase transitions and electrolyte leakage are the core factors driving changes in impedance spectra (Zhang, Ryyppö & Repo, 2002). At normal temperatures, the plant cell membrane exists in a liquid crystalline phase, characterized by high C_m_ and selective ion permeability. When the temperature drops to the membrane lipid phase transition point (typically between 0 °C and −10 °C), the membrane transitions into a gel phase, leading to decreased fluidity, reduced C_m_, and impaired function of membrane proteins (Repo et al., 1997). If the temperature continues to fall below freezing, the formation of intracellular ice crystals induces cellular dehydration and solute concentration, causing the plasma membrane to contract and resulting in a temporary increase in intracellular resistance (R_i_). However, when the temperature exceeds the cell’s tolerance limit, the membrane structure suffers irreversible damage or rupture, leading to the leakage of intracellular solutes, a significant decrease in R_e_, and a sharp decline in C_m_. For instance, in a freezing stress study on sapwood of Korean pine (*Pinus koraiensis*) and Simon poplar (*Populus simonii*) by Song et al. (2024), exposure to −40 °C caused R_e_ to increase several-fold compared to levels at −10 °C, while τ also showed a sharp rise within the deep freezing range of −70 °C to −80 °C. This clearly reveals the continuous structural damage process of the cell membrane caused by mechanical injury from ice crystals and electrolyte leakage. This series of continuous changes in electrical parameters visually documents the complete process of the cell membrane transitioning from stability, phase transition, and dehydration to final damage and leakage. It is noteworthy that cold-resistant plants can lower the membrane phase transition temperature by modulating lipid composition (*e.g*., increasing unsaturated fatty acids), thereby maintaining higher C_m_ and longer τ at low temperatures (Satyakam, Singh & Kumar, 2022; Zhou et al., 2025). For example, studies have found a significant positive correlation between higher R_e_ (indicating better membrane integrity) and longer τ (indicating greater membrane structural stability) in cold-resistant varieties, demonstrating delayed membrane phase transition and stronger maintenance of fluidity (Elwan et al., 2018; Zhang et al., 2022).

EIS parameters are closely linked to traditional cold-hardiness indicators. REL is a traditional metric for assessing cell membrane damage and ion leakage, and the R_e_ in EIS is closely related to it: a decrease in R_e_ corresponds to more ions entering the extracellular fluid, equivalent to an increase in REL (Sun et al., 2020). Numerous studies have confirmed the quantitative relationship between them (Thalhammer et al., 2020). For example, in studies on white pine (Li et al., 2009) and rose (Qian et al., 2021), the R_e_ measured by EIS showed high consistency with the cold hardiness results obtained by the conductivity method (EL method), and the variety rankings were similar. This indicates that EIS can serve as a non-destructive method for detecting electrolyte leakage, directly reflecting changes in membrane permeability through the dynamic variation of R_e_, and can provide more sensitive early time-series data. For instance, R_e_ begins to decrease within 1 h after freezing injury occurs, preceding visible injury symptoms (Repo et al., 2000).

Plant cold hardiness also involves reversible cellular dehydration and rehydration processes, a mechanism reflected in the dynamic changes of R_i_. When the temperature decreases, cellular dehydration and solute concentration lead to a temporary increase in R_i_; when the temperature rises again, water reabsorption by cells causes R_i_ to recover (Zhang, Li & Dong, 2010; Tiejun et al., 2024). Cold-resistant species exhibit a small, stable increase in R_i_ during cold acclimation, whereas sensitive varieties show greater fluctuations, indicating more severe disruption to cellular water balance (Neuner, 2014; Preißer & Bucher, 2021).

Furthermore, τ and its distribution width can reflect the heterogeneity of the cell population. When low temperature causes cell damage, population homogeneity decreases, leading to a broadening of the relaxation arc and a decrease in the CPE index *n* (Mancuso et al., 2004). Plants with poor cold resistance often exhibit impedance spectra with a small “radius” but a significantly depressed arc, characteristic of multiple relaxation processes, arising from the premature death of some cells while others remain intact. Conversely, cold-resistant varieties maintain more consistent and intact membrane characteristics. Therefore, combining relaxation behavior analysis helps in understanding the impact of low temperature on cell population heterogeneity. For example, in a study on stems of *Euonymus japonicus* by Li et al. (2010), it was found that during cold acclimation, stems treated with 5.0 mmol·L^−1^ salicylic acid showed a significant increase in τ compared to the control, and the intracellular resistivity (r_i_) also remained at a higher level. This phenomenon was interpreted as salicylic acid enhancing membrane lipid unsaturation and maintaining membrane structure and functional integrity, thereby effectively mitigating electrolyte leakage from cells and ultimately improving the plant’s cold hardiness.

In summary, since the pioneering research conducted by Repo and others, impedance spectroscopy provides a bridge between the physiological and biochemical processes of plant cold resistance and their electrical characteristics. Changes in electrical parameters, such as those caused by membrane phase transitions and damage, directly reflect the action of cold resistance mechanisms. EIS not only quantitatively evaluates cold resistance levels but also offers a unique perspective for analyzing these mechanisms. By comparing the variation patterns of EIS parameters across different genotypes, we can infer which physiological mechanisms, such as membrane stability or osmotic regulation, play a dominant role in cold resistance. This approach is invaluable for understanding the biophysical basis of cold resistance and guiding genetic improvement.

## The application progress of electrical impedance spectroscopy in the study of plant cold resistance

EIS and its imaging counterpart, EIT, have emerged as highly effective non-destructive testing techniques with significant potential in plant cold resistance research. EIS is capable of sensitively detecting changes in cell membrane integrity and ion permeability under low-temperature stress, while EIT can non-invasively visualize the two-dimensional/three-dimensional distribution of electrical conductivity within plant organs. This provides a unique opportunity to study the spatial dynamics of physiological processes under cold stress (Bottiglieri et al., 2020).

**Figure 2:**
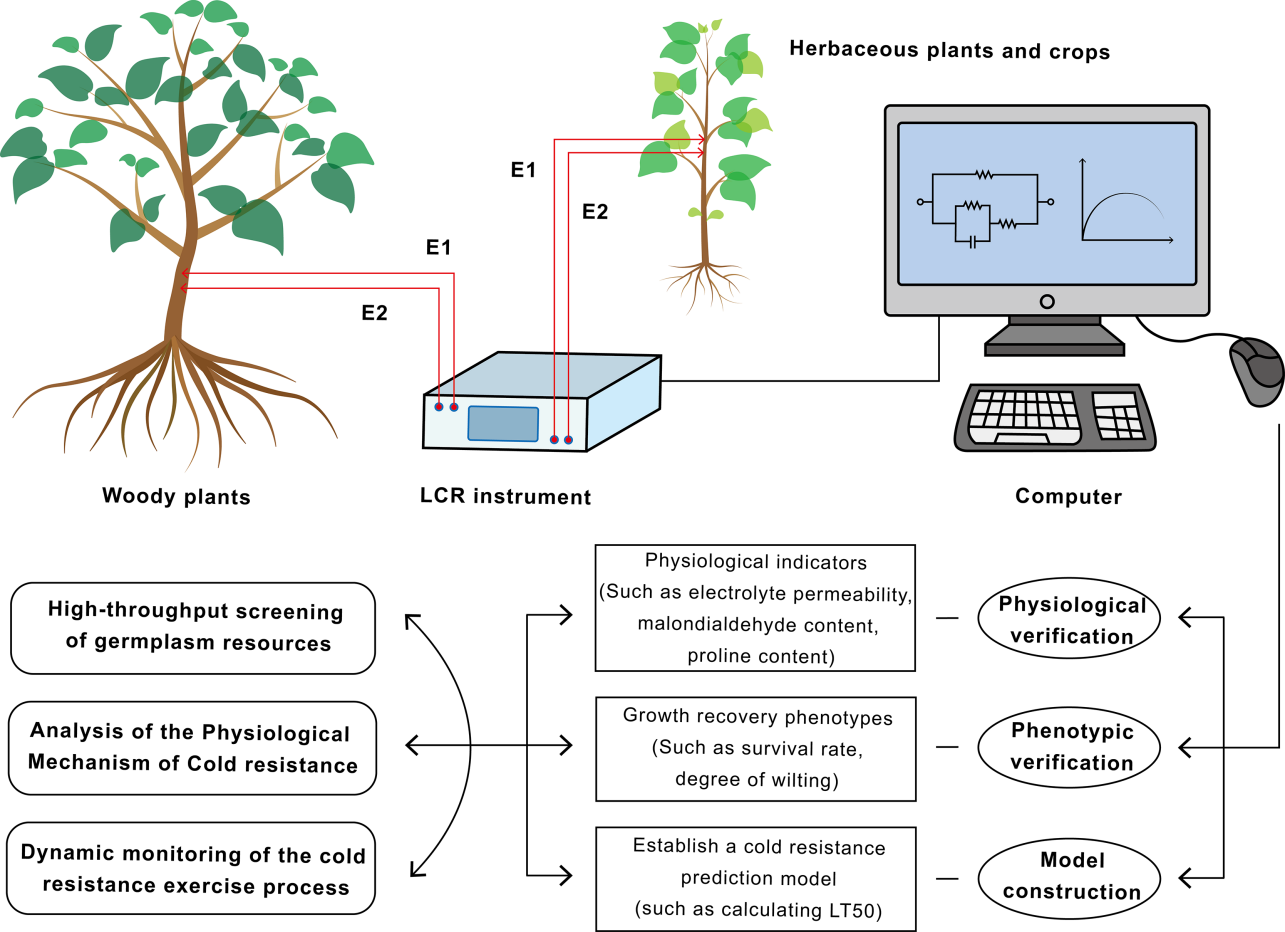
Flowchart of the application of electrical impedance spectroscopy (EIS) technology in plant cold resistance research and screening. This technology has achieved a closed loop from non-destructive measurement to mechanism research and breeding application.

In applications to woody plants, EIS technology has been successfully employed to evaluate cold hardiness across multiple tree species. For instance, a comparative study on important Australian ornamental species, *Callistemon* and *Grevillea*, demonstrated that models established based on EIS parameters could effectively differentiate the cold resistance levels of different species. The evaluation results showed a high degree of consistency with those obtained by the electrolyte leakage method (Mancuso et al., 2004). Subsequent research has further refined these applications. Work by Li et al. (2009), for example, revealed that in lacebark pine (*Pinus bungeana*), cold resistance of needles was more strongly correlated with R_i_, whereas in branches, R_e_ proved to be a better indicator. This finding laid the groundwork for EIS-based identification of cold hardiness in evergreen coniferous trees. For woody plants with complex tissue structures, EIS measurements often necessitate the use of inserted electrodes or detached branches (Fig. 2). Nevertheless, under controlled conditions, the derived parameters can still provide reliable information concerning cell membrane stability and plasma membrane leakage.

In the realm of herbaceous plants, EIS is similarly utilized to monitor physiological changes. One study employed EIS and the Double-Shell Model (DSM) to track physiological changes in lettuce leaves over a 16-h period. The research found that the impedance spectrum data of the lettuce leaves fitted well with the DSM model, and variations in the resistance-capacitance (R-C) parameters could reflect the physiological status of the leaves (Nouaze et al., 2022). A groundbreaking study achieved physical decoupling of the impedance responses from stems, roots, and soil by introducing a three-channel acquisition system combined with Cole-Cole model fitting. This approach was deployed for the large-scale screening of 80 wheat (*Triticum aestivum* L.) and 10 pecan (*Carya illinoinensis* (Wangenh.) K. Koch) accessions. This research not only validated the high correlation between EIS parameters (such as relaxation time and intracellular resistivity) and the classical electrolyte leakage method but, more importantly, demonstrated the capability of EIS to rapidly and reliably grade a large number of individuals with different genotypes. This provides a powerful tool for breeders screening for cold-resistant germplasm resources (Peruzzo et al., 2021). Research on subtropical crops with typically weaker cold resistance, such as rice (*Oryza sativa*), has shown that the leaf impedance spectrum undergoes significant dynamic changes under 4 °C cold treatment. An initial decrease in R_e_, followed by stabilization or even recovery, was consistent with changes in leaf REL. Furthermore, resistant and susceptible varieties exhibited distinct R_e_ change patterns, indicating that dynamic EIS monitoring can differentiate the cold resistance of crop varieties and serve as an auxiliary tool in breeding programs (Tiejun et al., 2024). Additionally, studies on herbaceous plants like alfalfa (*Medicago sativa* L.) and birdsfoot trefoil (*Lotus corniculatus* L.) revealed that the responses of resistance and reactance at different frequencies could provide new insights into understanding changes in structures like cell membranes and cell walls during cold acclimation (Liu et al., 2021). Overall, EIS is typically performed during the seedling stage or on vegetative organs (such as leaves and tubers). Its non-destructive and rapid characteristics make it ideally suited for large-scale screening, offering crop breeders the possibility to evaluate the cold resistance of numerous materials at an early stage.

To conduct more comprehensive assessments of plant cold tolerance, researchers often combine EIS with traditional methods and other emerging technologies to leverage their complementary advantages. For example, several studies have utilized EIS parameters in conjunction with Logistic models to calculate LT_50_. This EIS-LT_50_ approach has shown high consistency with results from the electrolyte leakage method in studies on the cold tolerance of species such as tea plant (*Camellia sinensis*), Chinese horse chestnut (*Aesculus chinensis*), and Chinese tulip tree (*Liriodendron chinense*) (Jin et al., 2012). Due to its non-destructive nature, this combined method allows for continuous monitoring of LT_50_ dynamics during the hardening process on the same sample, achieving a capability beyond the reach of traditional destructive methods. Some studies have correlated EIS results with changes in tissue microstructure. In rose branches, for instance, areas of high impedance precisely corresponded to vascular tissues exhibiting higher cell survival rates, indicating that EIS/EIT can spatially resolve tissue damage and provide complementary verification to microscopic observations. Furthermore, numerous studies integrate EIS with classic physiological and biochemical indicators, such as malondialdehyde content, soluble sugars, proteins, and protective enzyme activities. Analyzing the correlations between these indicators and EIS parameters deepens the understanding of the roles played by membrane lipid peroxidation, osmolyte accumulation, and reactive oxygen species scavenging in cold tolerance, thereby providing a more comprehensive interpretation of the physiological significance underlying EIS parameters (Qian et al., 2021). An increasingly promising direction involves the integration of EIS with molecular biology techniques like metabolomics and proteomics. For instance, an increase in R_i_ under cold stress in certain varieties might correlate with the accumulation of osmotic substances like proline. Transcriptomics could also be employed to identify gene expression differences influencing EIS parameters. Although direct integration of EIS with omics studies remains relatively uncommon, this interdisciplinary approach is anticipated to become an important future direction for in-depth analysis of cold resistance mechanisms (Qin et al., 2023).

EIS technology has become deeply integrated into both fundamental research and practical applications related to plant cold resistance. Its role is becoming increasingly comprehensive, spanning from the screening of cold-resistant varieties to the exploration of cold tolerance physiology and genetics. As EIS continues to converge and integrate with multiple disciplines, it is poised to play an even more critical role in plant stress resistance research.

## Technical advantages and challenges faced

EIS technology exhibits significant technical advantages in plant cold hardiness research. Its primary advantage lies in non-destructive testing capability, causing minimal damage to plants and thus enabling repeated, continuous monitoring of the same plant. This feature makes it particularly suitable for observing plant cold acclimation processes or tracking dynamic changes during freezing injury and recovery, while providing real-time data insights unattainable by traditional destructive methods (Cao et al., 2011). In terms of efficiency, EIS tests typically take only a few minutes to complete, which is much faster than time-consuming growth recovery experiments or separately processed electrolyte leakage methods. Combined with automated equipment, it enables simultaneous measurement of multiple samples, offering an ideal solution for high-throughput screening of large-scale germplasm resources and improved breeding efficiency (Jócsák, Végvári & Vozáry, 2019).

Moreover, EIS can acquire multiple electrical parameters in a single test, reflecting structural and functional information about cell membranes, electrolytes, and cytoplasm. Compared to traditional methods that only indicate ion leakage, this multi-parameter analysis more deeply reveals the intrinsic mechanisms of plant cold hardiness. For example, distinguishing changes in cell membrane capacitance and intracellular/extracellular resistance helps determine whether damage primarily occurs in membrane structures or cellular contents (Yang et al., 2023). Studies have confirmed that EIS parameters are closely correlated with classic cold hardiness indicators such as LT_50_ and survival rate, ensuring the biological significance and interpretability of the data it provides, which facilitates acceptance among breeders and physiological researchers (Prášil, Prášilová & Mařík, 2007). With the development of portable impedance measurement devices, EIS can be directly applied in field or storage environments to real-time monitor tree overwintering status or assess cold damage in horticultural crops. This on-site diagnostic capability, unavailable in many other analytical techniques, highlights its unique application value (Zhu et al., 2022).

Despite its prominent advantages, EIS faces numerous challenges in practical application and popularization. First, plant impedance characteristics vary with species, tissue type, and growth stage, requiring optimization of measurement protocols and equivalent model parameters for each new material. For instance, woody stems necessitate a two-electrode insertion method due to the presence of xylem, while succulent plant tissues may require adjusted upper frequency limits due to their high water content. Additionally, the biological interpretation of the same model parameter may differ across species, demanding cautious analysis (Hussain et al., 2021).

Second, factors such as plant water content, ambient temperature, and rhizosphere soil medium directly affect impedance measurements. In particular, the physical effect of temperature changes (increased resistance with decreasing temperature) must be distinguished from the physiological effects of low temperatures on biological membranes. Obtaining reliable results requires strict control or calibration of these environmental factors, posing challenges for EIS application under natural field conditions.

Third, regarding data analysis, it is crucial to select an appropriate equivalent circuit model and to accurately fit its parameters. However, the complex structure of plant tissues poses a major challenge for model selection (Michels, Weigand & Kemna, 2024). Even after model selection, parameter fitting may encounter convergence difficulties or unsatisfactory results. Furthermore, parameters such as CPE exponent lack direct biological counterparts, further increasing the complexity of result interpretation and requiring researchers to have a strong background in electrochemistry and mathematics.

Fourth, EIS generates large-scale datasets in the form of frequency functions, which require professional knowledge for feature extraction and interpretation. Currently, the academic community has not established standardized methods to correlate impedance spectrum changes with physiological states. For example, different studies may adopt varying frequency ranges and electrode configurations, making direct comparison of experimental results difficult. Establishing standardized data processing workflows and cold hardiness evaluation criteria is therefore key to promoting the wider application of EIS (Liu et al., 2021).

Finally, high-precision impedance analyzers are expensive, limiting their popularization in ordinary laboratories and field settings (Murbach, Hu & Schwartz, 2017). Additionally, measurement electrodes often need customization for specific samples, and consumable wear further increases application costs. Although its technical cost is lower than that of molecular biology methods, reducing equipment costs remains a necessary condition for large-scale commercial application.

In summary, while EIS technology offers significant advantages for plant cold resistance research, it must overcome several challenges to fully realize its potential. Addressing these issues requires methodological advancements and collaboration within the scientific community to establish standards, databases, and promote data sharing.

## Future perspectives

Looking ahead, EIS technology holds broad prospects in the field of plant cold hardiness research, and its evolution will be deeply integrated with instrument innovation, method standardization, and interdisciplinary collaboration. Developing plant-specific portable and intelligent EIS devices is a key step toward technological implementation and popularization. Handheld devices integrating micro impedance analysis chips and Internet of Things (IoT) technology will enable breeders to conduct rapid, non-destructive on-site assessment and remote monitoring of the cold hardiness of overwintering crops or fruit trees directly in the field, greatly improving breeding efficiency (Zhu et al., 2022; Choi et al., 2024). With the popularization of measurement equipment, promoting the standardization of measurement protocols and data analysis procedures will become an urgent task for the next phase. This requires the academic community to jointly develop unified measurement guidelines and establish an open and shared plant impedance spectrum database, thereby addressing the current challenge of poor data comparability and laying the foundation for big data-driven pattern mining. At the mechanistic research level, the application of EIS technology is evolving from phenotypic correlation to mechanistic elucidation. Future studies can use EIS to detect changes in the functional states of organelles such as chloroplasts and mitochondria under low temperatures. By linking with molecular biology techniques, changes in impedance parameters can be correlated with the expression of specific genes or the accumulation of metabolites, establishing causal relationships between electrophysiological responses and molecular events to systematically reveal the physiological and molecular mechanisms of plant cold hardiness (Cum et al., 2023).

Notably, machine learning (ML) and artificial intelligence (AI) provide powerful tools for addressing the analysis of massive EIS data (Kophamel et al., 2022; Van Haeverbeke, De Baets & Stock, 2023; Dapsance et al., 2023). As demonstrated in studies on plant water stress, AI models have successfully learned from EIS data to classify plant water status, and this “spectrum-phenotype” correlation prediction model is equally applicable to cold hardiness research. A recent study on maize seedlings has shown that combining EIS with machine learning algorithms such as support vector machines (SVMs) can accurately diagnose early cold stress damage with high precision, exceeding the accuracy of traditional indicators (Song et al., 2024). This fully demonstrates that using AI to extract features from complex impedance spectra that are beyond human visual recognition and constructing prediction models is expected to achieve automated, high-throughput, and intelligent identification of plant cold hardiness, ultimately providing core decision support for precision breeding. Finally, the application scope of EIS is expected to expand from single cold stress to the joint screening and discrimination of multiple abiotic stresses such as drought and salinity (Jócsák et al., 2010). By establishing a multi-stress response fingerprint database, EIS technology will facilitate the screening of elite germplasm with broad-spectrum stress resistance, offering strong technical support for addressing complex climate challenges and ensuring global food security.

## Conclusion

In summary, this review systematically collates the application progress of EIS technology in plant cold hardiness research, ranging from its successful cases in woody and herbaceous plants to its integration with electrophysiological models, traditional physiological indicators, and omics technologies. It fully demonstrates that EIS, as a tool with both theoretical depth and practical value, can provide a unique and sensitive observational window for understanding the physiological mechanisms of plant cold hardiness. To conclude, EIS technology has exhibited enormous potential to offer efficient and reliable methodological references for large-scale germplasm screening and breeding practices. With the continuous optimization of the technology itself, the establishment of standardized systems, and its in-depth integration with cutting-edge technologies such as artificial intelligence, EIS is expected to transform from an advanced laboratory research tool into a universal platform that runs through the entire chain of basic theoretical exploration and modern breeding, playing an increasingly crucial role in addressing global issues of environmental stress mitigation and food security assurance.

## Additional Information and Declarations

### Data Availability

The following information was supplied regarding data availability:

This manuscript is a literature review.
